# Thrombomodulin expression regulates tumorigenesis in bladder cancer

**DOI:** 10.1186/1471-2407-14-375

**Published:** 2014-05-28

**Authors:** Chun-Te Wu, Ying-Hsu Chang, Paul- Yang Lin, Wen-Cheng Chen, Miao-Fen Chen

**Affiliations:** 1 Department of Urology, Chang Gung Memorial Hospital at Keelung, Keelung, Taiwan; 2 Chang Gung University, College of medicine, Taoyuan, Taiwan; 3 Department of Urology, Chang Gung Memorial Hospital at Linko, Linko, Taiwan; 4 Department of Pathology, Chang Gung Memorial Hospital at Chiayi, Chiayi, Taiwan; 5 Radiation Oncology, Chang Gung Memorial Hospital at Chiayi, Chiayi, Taiwan

**Keywords:** Bladder cancer, Thrombomodulin, DNMT1, Epithelial-mesenchymal transition (EMT)

## Abstract

**Background:**

The identification of potential tumor markers will help improve therapeutic planning and patient management. Thrombomodulin (TM) is a sensitive urothelial marker. TM was reported to be one of the endogenous anti-metastatic factors and has diagnostic and prognostic values for the progression of carcinoma. In the present study, we examine the role of TM in bladder cancer.

**Methods:**

We studied the role of TM in tumor behavior and related signaling pathways *in vitro* using the human bladder cancer cell lines HT1376, HT1197, J82 and T24, and *in vivo* using animal models. We also selected clinical specimens from 100 patients with bladder cancer for immunohistochemical staining to evaluate the predictive capacity of TM in tumor invasiveness.

**Results:**

The data revealed that positive immunoreactivity for TM was inversely correlated with clinical stage and DNA methyltransferase 1 immunoreactivity. Decreased TM expression could predict the aggressive tumor growth and advanced clinical stage in bladder cancer. When TM was inhibited, tumor growth rate and invasion ability were augmented *in vitro* and *in vivo*. The underlying changes included increased cell proliferation, enhanced epithelial-mesenchymal transition (EMT) and angiogenesis. Moreover, inhibition of NF-κB activation significantly increased TM expression and attenuated tumor aggressiveness in bladder cancer.

**Conclusions:**

TM plays an important role in bladder cancer tumor aggressiveness *in vitro* and *in vivo* and is a clinically significant predictor that may represent a suitable therapeutic target for bladder cancer.

## Background

Urinary bladder cancer represents a spectrum of neoplasms, including non-muscle invasive, muscle invasive, and metastatic lesions. Approximately 70% of patients present with non-muscle invasive tumors, while the remaining 30% present with muscle-invasive bladder cancers. Despite good prognosis for patients with superficial disease, superficial bladder cancer has a very high rate of recurrence after treatment [1]. Of these recurrent tumors, 10–30% show progression in grade and stage [2]. Unlike other urological cancers, bladder cancer lacks clinically useful biomarkers for predicting disease stage and clinical outcome [3]. Therefore, molecular markers that can be used to stratify and identify the true malignant potential of a tumor and its response to specific therapies are required.

Thrombomodulin (TM), a type 1 transmembrane glycoprotein, is an anticoagulant factor [4,5] that mediates hemostatic function and regulates multiple functions important in inflammation and tumor promotion [6-9]. A potential role for TM in tumor biology was further suggested by studies showing that TM expression in tumor tissues correlated with a less advanced stage at diagnosis and a better prognosis for multiple cancers [6,10-12]. Anticoagulation, anti-inflammation, adhesion and proliferation have all been suggested as mechanisms mediating the effects of TM in tumors. Although TM expression in tumor cells has been suggested to limit the invasive potential and proliferation of tumor cells in certain types of cancers, its role in bladder cancer remains to be elucidated.

Chronic inflammation often precedes or accompanies a substantial number of cancers [13,14], and data from animal and human studies strongly support the hypothesis that chronic inflammation plays a role in bladder carcinogenesis [15,16]. TM has been considered as a sensitive, but nonspecific, marker of urothelial carcinoma [17,18]. Moreover, the anti-inflammatory role of TM has been clearly demonstrated *in vivo*[9,19,20], in which it modified the inflammatory response, maintained the integrity of cell-cell interactions, and reduced matrix degradation. Thus, TM might play a role in bladder cancer, even though the mechanisms underlying TM’s role remain unclear. Several factors are implicated in regulation of TM expression including DNA methylation and nuclear factor-Kappa B (NF-κB) [21,22]. We have previously reported that DNA methyltransferase1 (DNMT1) indicates more aggressive tumor growth and resistance to treatment in bladder cancer [23], and the activation of DNMT1 is enhanced by inflammatory cytokines [24]. Moreover, NF-κB is widely-recognized as a key regulator of the inflammatory responses, and plays an important role in various types of human cancers including bladder cancer [25-27]. Therefore, we proposed the inhibition of TM by NF-κB activation and DNMT1 might mediate in part the aggressive bladder tumor behavior.

Herein it is shown that decreased TM expression could predict the aggressive tumor growth and advanced clinical stage in bladder cancer. In addition, the link between TM signaling, the activation of NF-κB and DNMT1 in bladder cancer was demonstrated. The study highlight a potential role for TM as a molecular predictor and therapeutic target for bladder urothelial carcinoma.

## Methods

### Patient characteristics for immunohistochemical (IHC) staining

The Institutional Review Board of our Hospital approved the present study. Informed written consent was obtained from patients for the acquisition and storage of medical information and tissue specimens. A total of 100 patients with bladder TCC, 60 with muscle-invasive tumors and 40 with non-muscle-invasive tumors, were enrolled in the study. Formalin-fixed, paraffin-embedded tissues obtained by transurethral resection in the diagnosis were cut into 5-μm sections and mounted on slides for IHC staining. For histological evaluation of TM immunoreactivity, the staining was scored independently by two observers blinded to the clinical outcome; discordant scores were reviewed, and a consensus was reached. Positive IHC scoring was defined as > 10% positive tumor cells.

### Cell culture and reagents

Four human bladder cancer cell lines, HT1376, HT1197, T24 and J82, were obtained from the American Type Culture Collection (ATCC). We maintained the bladder cancer cell lines in Dulbecco’s modified Eagle’s medium supplemented with 10% fetal bovine serum. The TM silencing vector (the TM shRNA lentiviral transduction particles with puromycin resistance) and control vector (consisting of a non-effective scrambled shRNA cassette) were purchased from Santa Cruz Biotechnologies (Santa Cruz, CA, USA). Stable TM-silenced cancer cells were generated by transfecting bladder cancer cells with the TM silencing vector and selected by culturing in medium containing puromycin for 4 weeks. The DNA methyltransferase (DMNT) inhibitor 5-aza-2′-deoxycytidine (5-AZDC) and caffeic acid phenethyl ester (CAPE), a specific inhibitor of NF-κB, were obtained from Sigma (St. Louis, MO, USA).

### Ectopic and orthotopic tumor xenograft model

This study was carried out in strict accordance with the recommendations in the Guide for the Care and Use of Laboratory Animals as promulgated by the Institutes of Laboratory Animal Resources, National Research Council, U.S.A. The protocol was approved by the Committee on the Ethics of Animal Experiments of our Hospital. Eight-week-old female athymic nude mice were used as the xenograft tumor implantation model. In the ectopic tumor implantation model, 1 × 10^6^ tumor cells were subcutaneously implanted by injection into the dorsal gluteal region (five animals/group). Tumor size was measured every 3 days after implantation (day 0). The tumor volume was calculated assuming an ellipsoid shape. In the orthotopic tumor implantation model, we performed intravesicular instillation of cancer cells as described previously (five animals/group). The extent of orthotopic tumor invasion was measured after implantation at the indicated times. The effect of CAPE treatment was also investigated *in vivo*. In the treatment group, mice received intraperitoneal injection of 4 mg/kg CAPE solubilized in a saline vehicle solution containing 20% dimethylsulfoxide (DMSO) 3 times per week for 2 weeks starting on day 3. CAPE-treated mice were compared with the control group mice, which were treated with vehicle only.

### Cell migration and cell invasion assays

Cell invasion capacity was determined using the Cell Invasion Assay (Trevigen, Gaithersburg, MD, USA). The top chambers were pre-coated with basement membrane extract derived from the Engelbreth-Holm-Swarm (EHS) tumor provided in the kit. After incubation for 24 h, the number of cells in the bottom chamber was determined by measuring the fluorescent anion calcein released from the intracellular calcein acetoxymethylester. To validate experiments on cell migration, scratch assays were performed by drawing a 2-mm wide scratch across each cell layer using a pipette tip. The plates were photographed at the times indicated.

### Immunofluorescence (IF) staining

Cells were seeded onto glass coverslips at 5 × 10^4^ cells/ml in 6-well plates for IF staining with or without treatment. After treatment at the specified times, cells were fixed with 2% paraformaldehyde for 5 min and washed in phosphate buffered saline (PBS) with Tween-20 (PBST). The slides were incubated for 1 h at room temperature with antibodies against TM, E-cadherin and cleaved caspase 3, followed by incubation with a FITC-conjugated secondary antibody for 1 h and counterstained with DAPI to visualize nuclei.

### Real-time reverse transcription-polymerase chain reaction (RT-PCR)

Real-time RT-PCR was performed on RNA extracted from cell cultures. The primer sequences were as follows: (forward and reverse, respectively) 5′-TAACGAAGACACAGACTGCGA TT-3′ and 5′-CTAGCCCACGAGGTCAAGGT-3′ for TM. A β-actin primer set was used as a loading control. The optimized PCR was performed on an iCycler iQ Multicolor Real-Time PCR detection system. Significant fluorescent PCR signals from carcinoma tissue were normalized relative to the mean value of signals obtained from control samples.

### Statistical analysis

Data were presented as means ± standard error of the mean (SEM) in triplicate for each experiment, and the entire set of experiments was replicated at least twice. Significant differences between groups were assessed using the log-rank test. Significant differences between samples were determined using the Student’s t-test. All statistical tests were two-sided, with *P <* 0.05 indicating significance.

## Results

### Role of TM in tumor invasion and epithelial-mesenchymal transition (EMT)

**Figure 1:**
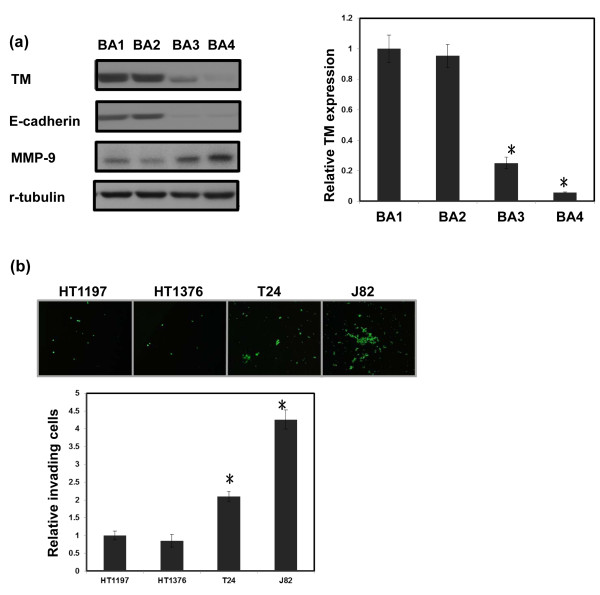
**Levels of TM in bladder cancer cell lines. (a)** Levels of TM were examined in HT1197, HT1376, T24 and J82 cell lines using RT-PCR and Western blot analyses. For real-time RT-PCR analysis, the y-axis represents the ratio of TM expression in four cancer cell lines relative to the expression in HT1197 cancer cells. **(b)** The invasive capacity of bladder cancer cells was evaluated by invasion assays. The results from representative slides are shown. The y-axis represents the ratio of invading cells detected in four bladder cancer cell lines normalized to that in HT1197 cancer cells. Data are expressed as the mean of three separate experiments ± SD; *P  <  0.05. (BA1 = HT1197; BA2 = HT1376; BA3 = T24; BA4 = J82).

**Figure 2:**
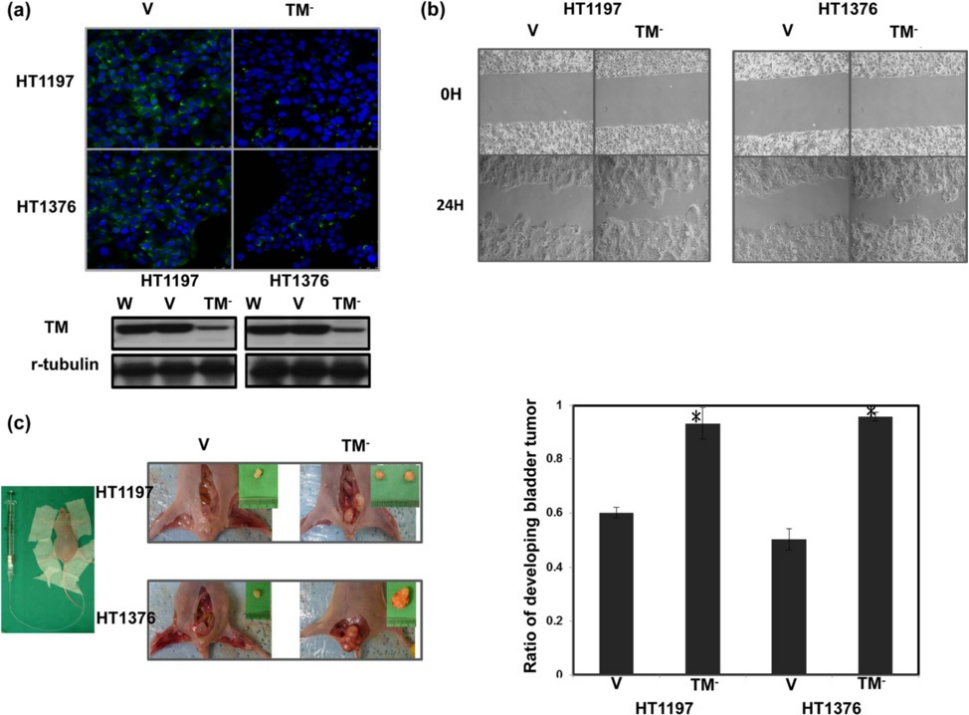
**Role of TM in tumor invasiveness. (a)** IF and Western blot analyses demonstrated the effects of the TM silencing vector on TM expression in HT1197 and HT1376 cells. Representative micrographs are shown (DAPI = blue; TM = green). TM levels were significantly decreased by the TM silencing vector compared with the control vector (CV). **(b)** The invasive capacity of bladder cancer cells expressing the TM silencing vector or CV was evaluated by migration scratch assays. The results from representative slides are shown. **(c)** The invasive capacity of bladder cancer cells was evaluated using murine orthotopic tumor implantation. The representative slides and quantitative data are shown. The y-axis represents the ratio of mice presenting intravesicular tumors normalized to that received orthotopic tumor implantation. The TM silencing vector increased the rate of tumor implantation in the bladder and was associated with larger tumor size. Data are expressed as the mean of three separate experiments ± SD; * *P  <*  0.05.

Differential expression of TM was observed in the four types of bladder cancer cells (Figure 1a), and expression of TM was negatively correlated with cell invasion *in vitro* (Figure 1b). To determine whether altering TM expression played a role in aggressive tumor growth, HT1197 and HT1376 bladder cancer cells, which have high TM expression levels, were transfected with a TM silencing or control vector. As shown in Figure 2a, the TM silencing vector significantly inhibited TM expression in both cell lines and augmented bladder cancer migration *in vitro* (Figure 2b). An orthotopic tumor implantation technique was used to examine the effect of TM silencing on tumor cell invasion *in vivo*. Mice received intravesical instillation of each bladder cancer cell line. After 21 days, intravesical tumors developed in 60% of mice instilled with HT1197 cells, in 93% with HT1197 cells + TM silencing vector, in 50% with HT1376 cells, and in 95% with HT1376 cells + TM silencing vector. As shown in Figure 2c, the TM silencing vector increased the rate of tumor implantation in the bladder and was associated with a larger tumor size.

**Figure 3:**
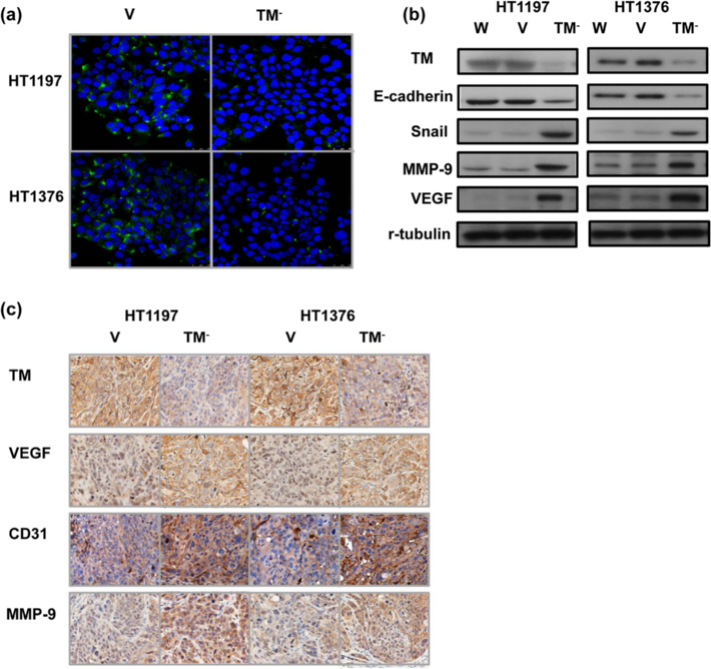
**Role of TM in EMT changes. (a)** Changes in E-cadherin expression were evaluated, and the representative micrographs are shown (DAPI = blue; E-cadherin = green). **(b)** Change in EMT-associated proteins in cells transfected with the TM silencing (TM^-^) or control vectors (CV). **(c)** Changes in VEGF, MMP-9, and CD31 expression in tumor xenografts were evaluated by IHC staining. The results from representative slides are shown.

EMT is a key event in invasiveness, and we determined whether it is the underlying mechanism responsible for the effects of TM on bladder cancer. Treatment with a TM silencing vector promoted EMT in bladder cancer cells, as determined by altered expression of E-cadherin and Snail (Figure 3a–b). Data from Western blots *in vitro* and IHC *in vivo* further revealed that the TM silencing vector resulted in higher expression of vascular endothelial growth factor (VEGF) and matrix metalloproteinase (MMP)-9 and increased angiogenesis (Figure 3b–c).

### Role of TM in tumor growth and related mechanisms

**Figure 4:**
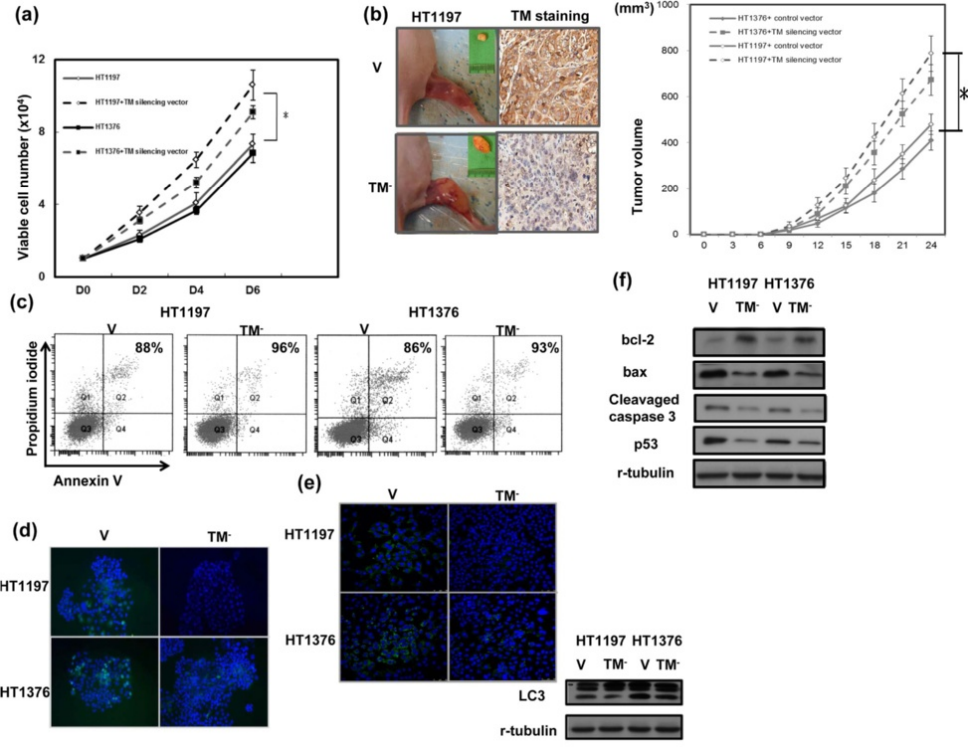
**Role of TM in tumor growth. (a)** Effects of the TM silencing vector on the proliferation rates of HT1197 and HT1376 cancer cells. The number of viable cells was counted after incubation for 2, 4, and 6 days. The y-axis represents the viable cell number. **(b)** Effects of TM inhibition on xenograft tumor growth. Each point represents the mean of three separate experiments ± SD; *, *P  <*  0.05. Expression of TM was also evaluated by immunohistochemical staining of xenografts. Representative slides are shown at × 400 magnification. **(c)** Flow cytometry using annexin V-propidium iodide (PI) staining for cell death rates in cells transfected with the TM silencing vector or control vector (CV). **(d)** Effect of TM silencing vector on apoptosis demonstrated by IF analysis. The results from representative slides are shown (DAPI = blue; cleaved caspase 3 = green). **(e)** Effect of TM silencing vector on autophagy demonstrated by IF analysis. The results from representative slides are shown (DAPI = blue; LC3 = green). The level of LC3 II was also examined by Western blot analysis in cells transfected with the CV or the TM silencing vector (TM^-^). **(f)** Effects of the TM silencing vector on the expression of apoptosis- and cell aging-related proteins evaluated by Western blot. Data points represent the mean of three separate experiments ± SD. *, *P  <*  0.05.

By counting the viable cell numbers over 6 days, we determined that the TM silencing vector significantly increased the proliferation rate of HT1197 and HT1376 cells (Figure 4a). Furthermore, using xenograft tumors, we observed that inhibition of TM resulted in an increased rate of tumor growth (Figure 4b). The data demonstrate that the TM silencing vector significantly augmented the growth rate of HT1197 and HT1376 bladder cancer cell lines. Tumor cells can be eliminated by apoptosis, necrosis, mitotic catastrophe, and premature senescence. Therefore, changes in the rates of cell death, apoptosis and autophagy were measured. The cell death rate decreased from 11.3 ± 1.4% to 4.3 ± 1.2% in HT1197 cells and from 13.7 ± 1.2% to 7.2 ± 1.3% in HT1376 cells after transfecting with the TM silencing vector (Figure 4c). The TM silencing vector noticeably decreased the rate of apoptosis, as determined by cleaved caspase 3 staining (Figure 4d). Furthermore, results from IF and Western blots using an antibody against LC3 demonstrated that the TM silencing vector resulted in a significant decrease in cell autophagy in HT1197 and HT1376 cells (Figure 4e). Moreover, Western blots showed that the rapid tumor growth induced by the TM silencing vector was associated with increased anti-apoptotic Bcl-2 and decreased p53 expression (Figure 4f).

### Level of TM in bladder cancer tissue

**Figure 5:**
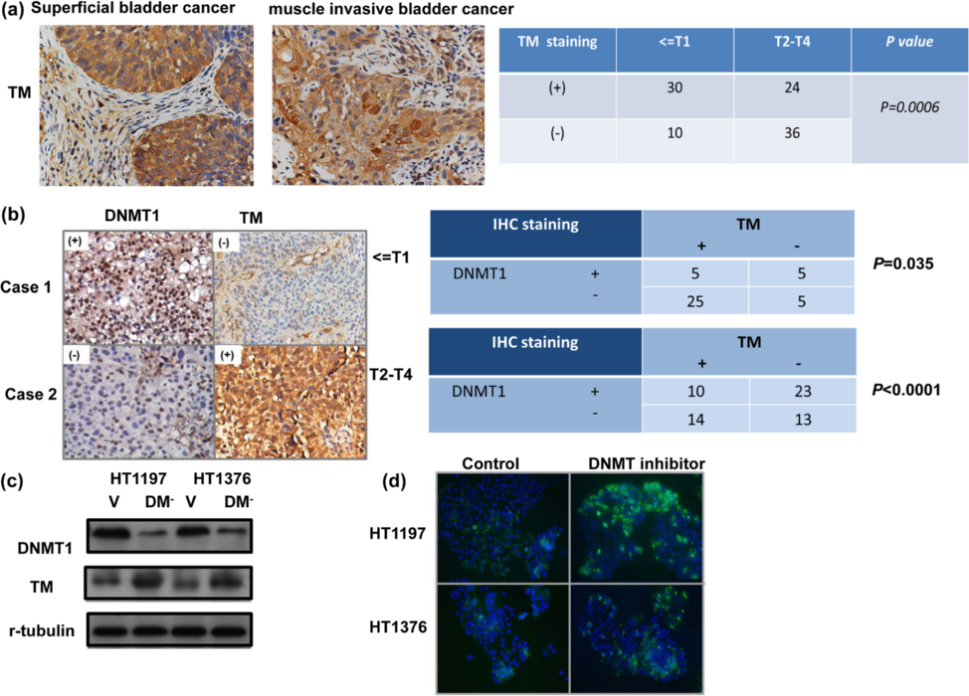
**Level of TM in bladder cancer. (a)** Immunohistochemical staining with anti-TM antibody on human bladder cancer specimens. Representative slides demonstrate that tumor cells showed TM-positive staining and that TM levels negatively correlated with clinical stage. **(b)** TM levels were negatively correlated with DNMT1 expression in human bladder cancer specimens (*P  <*  0.05). Representative slides of two selected tumor specimens demonstrating staining for both TM and DNMT1 are shown. **(c)** The effect of DNMT1 inhibition on the level of TM was examined by Western blots in cells transfected with the control vector (CV) and in cells transfected with the DNMT1 silencing vector (DM^-^). **(d)** Effect of DNMT inhibition on the level of TM was examined by IF analysis. The results from representative slides are shown (DAPI = blue; TM = green).

IHC staining of bladder tissue specimens from bladder cancer patients demonstrated that positive expression of TM inversely correlated with tumor invasion depth (Figure 5a). Positive staining for TM was evident in 40% (24/60) of T2-T4 bladder cancer tissues and 75% (30/40) of early stage tumors (CIS, Ta, or T1). We previously reported that higher DNMT1 levels were associated with aggressive tumor behavior and higher clinical stage in bladder cancers [22]. Thus, in this study, we further investigated the correlation between DNMT1 and TM expression. As shown in Figure 5b, there was a negative correlation between the expression of TM and DNMT1 in bladder cancer specimens. This result correlated with mRNA and protein levels *in vitro* demonstrating that inhibition of DNMT1 resulted in increased TM expression (Figure 5c).

### Effect of TM by CAPE on bladder cancer

**Figure 6:**
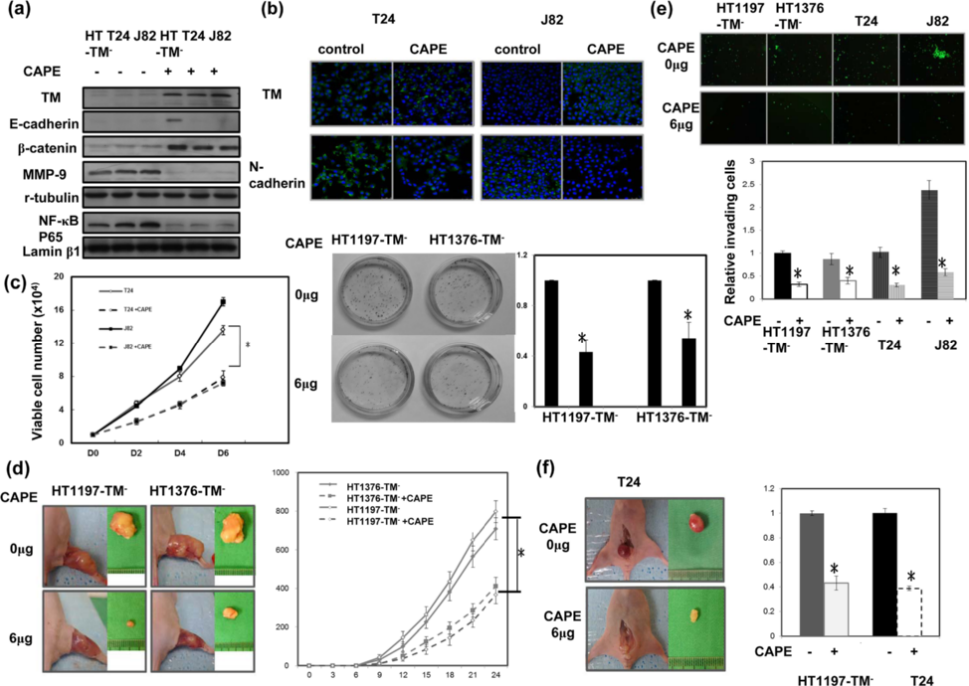
**Effects of CAPE on bladder cancer cells with lower TM expression. (a)** The effect of CAPE treatment on the level of TM, NF-κB activation and EMT-related changes was examined by Western blot using cells with lower TM expression in the presence or absence of 6 μg/ml CAPE for 48 h. **(b)** Effect of CAPE treatment on the level of N-cadherin was examined by IF analysis in cells with lower TM expression in the presence or absence of 6 μg/ml CAPE for 48 h. The results from representative slides are shown (DAPI = blue; N-cadherin = green). **(c)** Effects of CAPE on the proliferation rates of T24 and J82 cell lines were evaluated by viable cell counting and by colony formation in HT1197 and HT1376 cells with inhibited TM expression. **(d)** Effects of CAPE treatment on xenograft tumor growth. **(e)** Effects of CAPE treatment on the invasive capacity of bladder cancer cells expressing the TM silencing vector was evaluated by invasion assays. The results from representative slides are shown. **(f)** The invasive capacity of bladder cancer cells with or without CAPE treatment was evaluated by murine orthotopic tumor implantation. Representative slides and quantitative data are shown. Data are expressed as the mean of three separate experiments ± SD; * *P  <*  0.05.

NF-κB is a critical mediator of TM expression [21] and transcription of inflammatory cytokines [28], while CAPE significantly attenuated NF-κB activity and suppressed the expression of inflammatory cytokines in various cancers [29,30]. In the present study, we examined the effects of CAPE on TM expression and tumor aggressiveness of bladder cancer cells. CAPE significantly increased TM expression, which was associated with attenuated NF-κB activation (Figure 6a–b). The viability of bladder cancer cells after exposure to CAPE for 48 h was measured by absolute cell number counts and using a xenograft tumor growth model (Figure 6c–d), and CAPE was determined to decrease tumor growth *in vitro* and *in vivo*. We further examined whether CAPE attenuated the invasive capacity of bladder cancer cells, and found that it was suppressed by CAPE treatment. In addition to NF-κB activation, the expression of TM and the related EMT changes were also attenuated by CAPE. Moreover, intraperitoneal injection of 4 mg/kg CAPE significantly reduced the incidence of developing intravesical tumors (Figure 6f).

## Discussion

TM, an endothelial thrombin receptor, is a sensitive urothelial marker expressed in 48–90% of urothelial carcinomas [17,18]. Although TM has been suggested possessing prognostic value in some cancers [6,10-12], the predictive role of TM in bladder cancer requires further investigation. Therefore, we evaluated tumor suppression role of TM in bladder cancer in the present study. The data obtained from cellular experiments using bladder cancer cell lines revealed that the expression of TM was inversely correlated with the invasive ability. To investigate whether TM was responsible for the less aggressive behavior of bladder TCC, TM was suppressed in bladder cancer cells by stable transfection with a silencing vector. We found that the TM silencing vector significantly augmented the invasive ability of cells detected in cellular invasion assays and mouse orthotopic models. The molecular and phenotypic changes involved in EMT appear to be functionally relevant to the invasive characteristics of epithelial tumors including bladder cancer [31,32]. At the molecular level, EMT is characterized by loss of E-cadherin, a hallmark of EMT, and increased expression of invasion-related factors [33]. This loss of E-cadherin was consistently observed at sites of EMT in different human cancers and with increased tumor cell invasiveness. We demonstrated that silencing TM expression abolished the expression of E-cadherin, associated with increased Snail, VEGF and MMP-9 expressions. Snail has been reported to represses transcription from the E-cadherin promoter and promotes tumor cell metastasis [34,35]. In addition, increased VEGF and MMP-9 expression correlated with EMT changes and poor prognosis of bladder cancer [36,37]. Angiogenesis is one of the mechanisms that promote tumor progression, and it involves CD31-mediated endothelial cell-cell interactions [38]. Our *in vivo* data revealed that TM silencing augmented tumor invasiveness, which was associated with increased MMP-9, CD31 and VEGF expression in tumors. On the basis of these findings, changes in EMT might be responsible for reduced invasiveness in TM-positive urothelial cancers.

In addition to EMT changes, we demonstrated that TM silencing resulted in increased bladder tumor growth *in vitro* and *in vivo*. Our *in vitro* experiments demonstrated that inhibition of several types of cell death, including apoptosis and cellular autophagy, were responsible for the aggressive tumor growth induced by inhibiting TM. Decreased cell death induced by the TM silencing vector was associated with increased anti-apoptotic Bcl-1 expression and decreased p53 expression.

Identification of potential molecular markers has important implications for the development and selection of molecular targeting in cancer therapy. Our *in vitro* and *in vivo* data revealed that loss of TM was associated with more aggressive tumor growth. Accordingly, we proposed that this loss of TM might be a clinically relevant characteristic linked to disease progression in bladder cancer. To test this hypothesis, we examined the expression of TM in bladder cancer tissue using IHC and found that it was expressed in 54% of these bladder cancer specimens. Positive staining for TM was preferentially associated with lower cancer stages relative to muscle-invasive bladder TCC (40% of T2–T4 bladder cancer tissues vs. 75% in Ta–T1). Our study established that the expression of TM in muscle-invasive human bladder cancer is down-regulated in comparison with superficial bladder cancer.

TM is reported to be expressed at the cell surface in normal cells of epithelial origin, while TM staining was not found in cells undergoing transformation into a malignant phenotype [39]. Silencing of the TM gene promoter has been implicated in the down-regulation of TM synthesis, [40]. Aberrant DNA methylation plays a key role in carcinogenesis, leading to the epigenetic silencing of the expression of tumor-suppressor genes. Moreover, the expression of TM was restored after treatment with DNMT inhibitor in certain types of cancer cells [22,41]. We previously reported [23] that DNMT1 could be a significant clinical predictor of bladder cancer. Accordingly, we further investigate the link between TM expression and DNMT1 in the present study. A negative correlation between TM-positive samples and nuclear staining for DNMT1 was found using IHC. We further examined the relationship between TM and DNMT1 by regulating DNMT1 expression. The mRNA and protein levels revealed that inhibition of DNMT1 suppressed TM expression. We therefore suggest that inhibition of TM expression might be responsible for aggressive tumors in DNMT1-positive bladder cancer.

**Figure 7:**
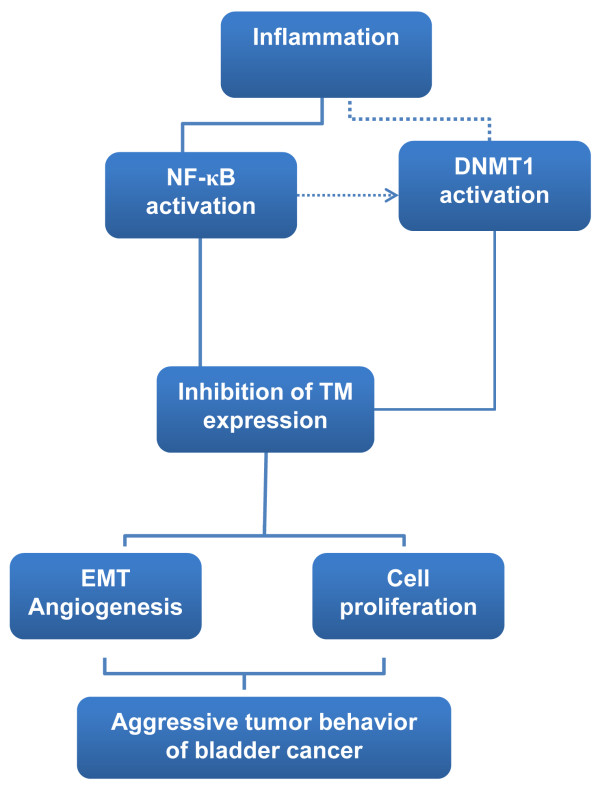
TM signaling pathway in bladder cancer.

Proinflammatory cytokines may be one of the mechanisms underlying the notion that chronic inflammation facilitates tumor progression [15]. Studies have identified that DNMT1 expression may be directly altered by pro-inflammatory cytokines such as IL-6. Many of the transcriptional effects of inflammatory cytokines are mediated by activation of NF-κB [25]. In cancer cells, NF-κB is often activated aberrantly, promoting the invasion, metastasis, and survival of these cells. Indeed, NF-κB overexpression was associated with poor prognosis in various malignancies, including bladder cancer [26,27]. The regulation of TM by inflammatory mediators has been studied by several groups, who concluded that NF-κB is a critical mediator of TM expression by inflammatory cytokines [21]. Therefore, we propose that inhibition of NF–κB activation may be a candidate strategy to regulate TM expression and tumor promotion for bladder cancer. CAPE, an active anti-inflammatory component of propolis, is a specific inhibitor of NF-κB [42]. CAPE has been reported to significantly inhibit the activation of NF-κB and attenuate proinflammatory cytokine production in cancer cells [29,30,43]. In the present study, we found that attenuated NF-κB activation by CAPE treatment was associated with increased expression of TM in bladder cancer cells. Our *in vitro* and *in vivo* data demonstrated that CAPE obviously inhibited tumor growth and impaired invasive ability associated with increased TM and attenuated NF-κB activation and EMT changes. By the data, we suggested that regulation of TM expression is critical in tumor aggressiveness and prognosis of bladder cancer. We outlined the main signaling pathways that are thought to link inflammation and TM signaling to the promotion of bladder cancer (Figure 7).

## Conclusion

Our *in vitro* and *in vivo* data indicate that TM plays an important role in bladder cancer tumor aggressiveness. Moreover, TM is a clinically significant predictor that might be partially responsible for aggressive tumors in DNMT1-positive bladder cancer. CAPE could be a promising therapeutic agent for bladder cancer that inhibits NF-κB activation and reverses the response induced by TM inhibition. Our study has established a foundation for future studies on the role of TM in bladder cancer progression and the therapeutic potential of TM in bladder cancer treatment.

## Competing interests

The authors declare that they have no competing interests.

## Authors’ contributions

CTW performed the study, and drafted the manuscript. YHC conceived of the study and participated in its design and coordination. PYL helped in histology and IHC staining. CFW and WCC conceived of the study and assisted in editing of manuscript. MFC performed the study, participated in its design, and coordination and assisted in editing of manuscript. All authors read and approved the final manuscript.

## Pre-publication history

The pre-publication history for this paper can be accessed here:

http://www.biomedcentral.com/1471-2407/14/375/prepub
